# Single-Agent and Associated Therapies with Monoclonal Antibodies: What About Follicular Lymphoma?

**DOI:** 10.3390/cancers17101602

**Published:** 2025-05-08

**Authors:** Gabriella Cancemi, Chiara Campo, Santino Caserta, Iolanda Rizzotti, Donato Mannina

**Affiliations:** 1 Hematology Unit, Oncology-Hematology Department, Azienda Ospedaliera Papardo, 98158 Messina, Italy; gabriellacancemi@aopapardo.it (G.C.); chiaracampo@aopapardo.it (C.C.); iolandarizzotti@aopapardo.it (I.R.); 2 Hematology Unit, Department of Human Pathology in Adulthood and Childhood “Gaetano Barresi”, University of Messina, Via Consolare Valeria, 98125 Messina, Italy; santino.caserta@polime.it

**Keywords:** monoclonal antibodies, rituximab, obinutuzumab, bispecific antibodies, mosunetuzumab, follicular lymphoma

## Abstract

Monoclonal antibodies (mAbs) have become a cornerstone in the treatment of follicular lymphoma (FL), offering highly specific therapeutic targeting that enhances efficacy while minimizing systemic toxicity. Their mechanisms of action include antibody-dependent cellular cytotoxicity (ADCC), complement-dependent cytotoxicity (CDC), and direct apoptotic signaling, effectively mediating malignant B-cell depletion. Anti-CD20 mAbs, such as rituximab and obinutuzumab, have significantly improved progression-free survival (PFS) and overall survival (OS), establishing immunochemotherapy as the standard of care for FL. However, the emergence of treatment resistance, often characterized by CD20 antigen downregulation or immune escape, has prompted the development of next-generation mAbs with enhanced effector functions. Bispecific antibodies (BsAbs), which simultaneously engage CD20-expressing tumor cells and CD3-positive cytotoxic T cells, have emerged as a novel immunotherapeutic strategy, redirecting T-cell activity to eliminate malignant B cells independently of major histocompatibility complex (MHC) antigen presentation. Additionally, antibody–drug conjugates (ADCs) offer a targeted cytotoxic approach by delivering potent chemotherapeutic payloads directly to tumor cells while limiting off-target effects. The integration of mAbs with immune checkpoint inhibitors and immunomodulatory agents is further enhancing treatment outcomes by overcoming immunosuppressive mechanisms within the tumor microenvironment. Despite these advancements, challenges remain, including optimizing the treatment sequence, mitigating immune-related toxicities—particularly cytokine release syndrome (CRS)—and identifying predictive biomarkers to guide patient selection. As the role of monoclonal antibodies continues to expand, their integration into therapeutic regimens is transforming the management of FL, paving the way for chemotherapy-free treatment approaches and long-term disease control. This review provides an updated overview of mAbs therapies for FL, emphasizing the advances brought by BsAbs and ADCs toward more tailored and effective treatments.

## Simple Summary

Monoclonal antibodies are crucial in treating follicular lymphoma, offering targeted therapy with reduced toxicity. Anti-CD20 monoclonal antibodies like rituximab and obinutuzumab have improved survival rates, making immunochemotherapy the standard. However, resistance due to CD20 downregulation or immune escape has led to next-generation drugs, such as bispecific antibodies and antibody–drug conjugates. These innovations, combined with immune checkpoint inhibitors, enhance outcomes. Despite progress, several challenges persist, including the optimal sequence of chemoimmunotherapy, bispecific antibodies, and CAR-T cell therapy; the management of immune-related toxicities, cytokine release syndrome and neurotoxicity; and the identification of predictive biomarkers, such as EZH2 mutations, minimal residual disease (MRD) status, and MHC class II expression, to better personalize treatment approaches.

## 1. Introduction

### 1.1. General Background

Monoclonal antibodies (mAbs) can be produced to bind to virtually any suitable target, and, currently, it is possible to have human/humanized mAbs, minimizing the risks originally associated with previous molecules [1]. The other benefit of mAb therapy is that it relies less on the patient’s immune response than vaccines, which is important in patients receiving immunosuppressive treatment. mAbs are laboratory-made proteins that act like antibodies and attack specific epitopes on antigens. They are developed from a cell lineage created by cloning a unique white blood cell. Modern medicine is further revolutionizing toward personalized “tailored” therapy adapted to individually specific disease characteristics [2]. Cancer research has long aimed to create immunotherapies that reroute and strengthen the anti-tumor capabilities of the immune system, and we are lucky to currently have a number of such agents available to treat B-non-Hodgkin lymphoma (NHL) [3,4,5]. Follicular lymphoma (FL) is an indolent B-cell neoplasm originating from germinal center cells, accounting for approximately 20–30% of non-Hodgkin lymphomas. Although generally associated with prolonged survival, it follows a relapsing course and carries a non-negligible risk of transformation into aggressive histologies [6]. This review aims to update the state of the art on the use of mAbs in FL, with a particular focus on the therapeutic innovations introduced by bispecific antibodies (BsAbs) and antibody–drug conjugates (ADCs), which are paving the way for more personalized and effective treatment strategies.

### 1.2. A Bit of History About Monoclonal Antibodies

In the following sections, we are going to focus on the first successful monoclonal antibodies used in the treatment of cancer.

#### 1.2.1. The First Monoclonal Antibody: Rituximab

Rituximab’s approval signaled a paradigm shift in cancer treatment by introducing the idea of targeted immunotherapy, which avoided many of the toxicities associated with cytotoxic chemotherapy [7]. It targets the CD20 antigen present on the surface of normal mature B lymphocytes and on neoplastic B cells of most lymphomas [8]. Following the binding of the Fab domain of rituximab to the target antigen, the action on B cells occurs through various mechanisms, the main ones of which are antibody-dependent cell-mediated cytotoxicity (ADCC), complement-dependent cytotoxicity (CDC), and effects directly induced by the binding of the antibody to CD20, including apoptosis [9]. Rituximab changed the way researchers approached drug development and opened the field of antibody-based drugs in oncology; in fact, it is the first successful monoclonal antibody in cancer therapy [7,10].

#### 1.2.2. The First Antibody–Drug Conjugate: Gemtuzumab Ozogamicin

Rituximab kills cancer cells using ADCC or CDC, which provide precise targeting of specific cell types but are less effective at causing cell death than cytotoxic agents. So, researchers concentrated on creating antibody-based drugs with a higher sensitivity to cancer cell killing by combining the precision targeting provided by an antibody with the potent killing offered by cytotoxic drugs. As a result, the first antibody–drug conjugate (ADC) was created to treat acute myeloid leukemia (AML) [11]. In the 1980s, AML had a poor outlook, especially for older or relapsed patients. Gemtuzumab ozogamicin emerged from two discoveries: antibodies targeting CD33 on AML cells and calicheamicin, a potent bacterial toxin. Though too toxic on its own, calicheamicin was safely delivered by linking it to the anti-CD33 antibody, forming an ADC [12]. This ADC specifically targeted and killed CD33+ AML cells. Preclinical studies confirmed its effectiveness and manageable toxicity. Gemtuzumab ozogamicin led to the rise of ADCs, with 12 now approved for cancer treatment and a 13th under review [13]. By 2026, ADC sales are expected to exceed USD 16.4 billion globally [14].

#### 1.2.3. The First Bispecific Antibody: Blinatumomab

Antibodies can activate immune cells like natural killer (NK) cells and macrophages through Fc receptors, but they cannot directly activate T cells, which lack these receptors [15]. Since T cells are potent cancer-killing cells, researchers sought ways to engage them. In 1982, the T cell receptor (TCR) was described, enabling T cells to recognize antigens. This led to the creation of bispecific T-cell engagers (BsAbs), which link T cells to cancer cells by binding both the TCR-CD3 complex and a tumor antigen [16]. In 2000, researchers in Germany developed the first BsAb, combining scFvs targeting CD3 and CD19. Produced in Chinese hamster ovary (CHO) cells, this antibody—later named blinatumomab—was tested in chimpanzees, showing strong T-cell activation and minimal side effects. Human trials followed, showing success in treating B-cell lymphomas and pre-B acute leukemia. In recent years, 12 BsAbs have received FDA and EMA approval, surpassing monoclonal antibody approvals due to their enhanced cytotoxicity and ability to target cells with low antigen expression. BsAbs also offer advantages over CAR-T cell therapy, including simpler logistics, off-the-shelf availability, and compatibility with other treatments, making them especially promising for broader use in both developed and developing countries [17].

## 2. Follicular Lymphoma

### 2.1. From Diagnosis to Risk Stratification

FL is the second most common type of NHL after diffuse large B-cell lymphoma (DLBCL), accounting for nearly 30% of all lymphomas, and is the most common subtype of clinically indolent NHL [18]. FL primarily affects adults and is uncommon in individuals under the age of 18. At the time of diagnosis, the median age of patients is approximately 65 years [19]. Each year, histologic progression from FL to DLBCL is observed in about 2% to 3% of cases [20,21].

The molecular mechanisms underlying this transformation have been increasingly elucidated over recent years. Transformed FL (tFL) arises through a complex multistep evolutionary process in which an indolent FL acquires additional genetic and epigenetic alterations that confer aggressive clinical behavior [22]. The earliest events in FL pathogenesis, notably the t (14;18) (q32;q21) translocation leading to constitutive BCL2 overexpression, create a background of impaired apoptosis but are not sufficient for malignant progression [23]. Transformation is driven by the sequential acquisition of further lesions that impact key cellular programs, such as proliferation, the DNA damage response, immune escape, and epigenetic regulation [22,24].

Among the most consistently implicated alterations in the transformation process are mutations in TP53, deletions in CDKN2A, and MYC rearrangements, each contributing to deregulated cell cycle control and genomic instability [22,25]. Furthermore, mutations in chromatin regulators such as CREBBP, EP300, and EZH2 are frequent and reshape the epigenetic landscape to favor aggressive behavior [22,26].

Primary diffuse large B-cell lymphoma (PDO) arising from a follicular background shares many of these alterations, although it often presents de novo with an aggressive clinical phenotype [27]. High-throughput sequencing studies have revealed that both tFL and PDO frequently harbor disruptions in immune regulation pathways, notably affecting genes like B2M and CIITA, and leading to immune evasion [24]. Transcriptomic analyses demonstrate a shift toward an activated B-cell-like (ABC) molecular profile during transformation, which is associated with an inferior prognosis [28].

Clonal evolution patterns in tFL suggest that transformation may emerge from either dominant clones or pre-existing minor subclones harboring aggressive genetic lesions, as recently confirmed by comprehensive genomic studies [22,25]. Understanding the molecular pathogenesis of transformation is critical for early risk stratification and for developing strategies aimed at intercepting the evolution toward aggressive disease.

This lymphoma is a slow-growing B-cell lymphoproliferative disorder that is often diagnosed at advanced stages, with fewer than 10% of patients being in stage I-II at the time of diagnosis. Approximately 70% of patients exhibit bone marrow involvement at diagnosis [29]. FL originates from germinal center B cells and is predominantly characterized by the presence of the t (14;18) (q32;q21) translocation in about 80% of cases, which leads to the overexpression of B-cell lymphoma 2 (BCL2), an anti-apoptotic protein. Approximately 5% of FL cases exhibit regulatory mutations in B-cell lymphoma 6 (BCL6) [30,31].

The morphological evaluation of a lymph node excisional biopsy plays a fundamental role in diagnosing FL. The tissue structure of FL generally exhibits a follicular growth pattern, but unlike reactive lymph nodes, the arrangement of centrocytes and centroblasts appears disorganized and lacks a clear pattern [32]. Histologically, FL is classified into grades 1 to 3, primarily based on the number of centroblasts present. Grade 3 is further divided into subtypes 3A and 3B. Grade 3B FL represents a distinct pathological and biological entity within the spectrum of FL, with features intermediate between indolent FL and aggressive large B-cell lymphomas [22,25].

Histologically, FL 3B is characterized by a uniform proliferation of centroblasts arranged predominantly in a follicular pattern, often with disrupted or confluent follicles [22,25]. The near-complete absence of centrocytes distinguishes it sharply from grades 1–3A FL [22].

Immunophenotypically, CD10 expression is frequently diminished or absent [33], and BCL2 protein expression is variable, reflecting the lower prevalence of the canonical t (14;18) translocation [34].

Indeed, molecular studies have shown that less than half of FL 3B cases harbor BCL2 rearrangements, a striking difference from classical FL [34]. Instead, FL 3B is enriched for genetic alterations typically associated with high-grade B-cell lymphomas, including BCL6 rearrangements, TP53 mutations, MYC aberrations, and mutations affecting chromatin-modifying genes such as CREBBP and EZH2 [26,35]. Next-generation sequencing analyses confirm that FL 3B harbors a mutational landscape closer to transformed FL and de novo DLBCL, particularly affecting pathways of immune evasion and DNA damage repair [36]. Gene expression profiling further reveals that FL 3B cases frequently display an ABC molecular signature rather than the germinal center B-cell–like (GCB) profile predominant in lower-grade FL [28].

Recognizing grade 3B as a distinct biological entity is essential, requiring an integrated diagnostic approach combining morphology, immunohistochemistry, cytogenetics, and molecular profiling.

Notably, grade 3B FL is classified and managed similarly to DLBCL due to its more aggressive behavior [7]. Grades 1–2 FL are typically associated with an indolent clinical course, whereas the prognosis for grade 3A remains debated, with some studies indicating a course similar to grades 1–2 and others suggesting a more aggressive progression [37,38].

Nearly all cases exhibit positivity for CD19, CD20, CD22, CD79a, and CD10 (found in approximately 60% of cases), while CD5, CD43 (in most cases), and CD11c are not expressed. CD23 expression varies, but it is generally negative. In grades 1–2 FL, the Ki-67 proliferation index is frequently below 20% [30,31]. The BCL2 protein is strongly expressed in almost all patients with grade 1 and 2 subtypes. BCL6 is present in at least some neoplastic cells across all FL tumors [39]. BCL6 rearrangements are more frequently observed in higher-grade FL, particularly in grade 3B, and are associated with a more aggressive disease course [40]. Molecular genetic studies indicate that the KMT2D mutation is detected in approximately 89% of tumors [41].

The underlying causes of FL remain largely unclear in most patients, although several risk factors are currently being studied [42]. Over the past two decades, significant advancements have been made in understanding the disease’s biology. Recurrent mutations affecting histones, the JAK-STAT pathway, and NF-kappa B signaling are frequently observed, along with early driver mutations in chromatin regulatory genes such as CREBBP, EZH2, and KMT2D [43]. The tumor microenvironment is highly heterogeneous, characterized by variations in T-cell subsets and MHC expression on FL cells, which significantly influence tumor behavior. Notably, gene expression patterns within the tumor microenvironment appear to be more predictive of patient outcomes than the lymphoma’s genetic profile [36,44]. Furthermore, multiple clones with distinct mutational alterations may coexist within the same patient [45]. This combination of genetic, molecular, and clonal heterogeneity, along with the impact of the microenvironment, make the development of precision medicine for FL particularly challenging [46,47].

The Follicular Lymphoma International Prognostic Index (FLIPI) [48] and its updated version, FLIPI2 [48,49], are the most effective tools for estimating the prognosis of newly diagnosed patients. The original FLIPI model primarily focuses on overall survival (OS), whereas FLIPI2 is designed to predict progression-free survival (PFS). The most advanced risk assessment model currently available is M7-FLIPI [50], which integrates the mutational status of seven key genes (EZH2, ARID1A, MEF2B, EP300, FOXO1, CREBBP, and CARD11) to refine the risk stratification (Table 1). Another prognostic model, known as the PRIMA-Prognostic Index (PRIMA-PI), is based on just two straightforward factors: bone marrow involvement and β2-microglobulin levels [48,49,50,51,52,53]. Several studies have assessed the prognostic significance of end-of-treatment fluoride-deoxyglucose positron emission tomography (FDG-PET) scans, showing a strong association with both the PFS and OS of patients with FL [54,55,56]. The most reliable indicator of the OS of FL patients is disease progression within 24 months (POD-24) following initial treatment. Among patients treated with R-CHOP, the 5-year OS rate was 50% in those with early disease progression compared to 90% in those who did not experience early progression [57,58,59]. The staging process for FL relies on findings from imaging studies through computed tomography (CT) and FDG-PET, and a bone marrow examination. It follows the Lugano classification, which is an updated version of the Ann Arbor staging system. Lymphoma is primarily categorized into two main stages: limited stage, which includes stages I and II, and advanced stage, comprising stages III and IV [60].

### 2.2. Treatment of Follicular Lymphoma

The treatment of FL varies based on the disease stage, tumor burden, and patient characteristics, with distinct therapeutic strategies for each group [32]. In patients with localized disease (stages I-II) and low-risk features, involved-site radiation therapy (ISRT) is the preferred treatment option. The standard dose of 24 Gy in 12 fractions has been associated with 10-year OS rates ranging from 60% to 80%. While the addition of rituximab or chemotherapy may improve PFS, no significant impact on OS has been demonstrated [59,60]. In selected cases, particularly in asymptomatic patients without bulky disease, an active surveillance strategy without immediate treatment may be a reasonable option [61]. In contrast, for bulky disease, systemic immunochemotherapy approaches are preferred, using regimens similar to those employed for advanced-stage disease.

The FIL MIRO (Molecularly Immuno-Radiotherapy Oriented) study tested the effects of consolidation with an anti-CD20 monoclonal antibody used after radiotherapy, on patients with minimal residual disease and therefore with traces of the disease in the blood and marrow after radiotherapy treatment. The primary objective of the study, obtaining a negative minimal residual disease (MRD) after consolidation, was largely achieved; the use of the monoclonal antibody, in fact, allowed 92% of patients to turn negative compared to the 50% objective set by the researchers. Unlike previous consolidation strategies based on rituximab, the FIL MIRO study employed ofatumumab, a fully human anti-CD20 monoclonal antibody that targets a different epitope of CD20 and demonstrates enhanced complement-dependent cytotoxicity [62]. This choice was made based on the hypothesis that ofatumumab could offer superior activity in eradicating residual disease. However, although MRD negativity rates were high, the long-term clinical benefits compared to rituximab-based approaches remain to be fully established [62].

The negative MRD condition, however, was obtained and significantly correlated with a better prognosis, confirming the validity of the principle of treating patients with positive MRD [62].

For grade 3B FL, which exhibits a more aggressive behavior similar to DLBCL, the recommended treatment is combination chemotherapy with R-CHOP (rituximab, cyclophosphamide, doxorubicin, vincristine, and prednisone) or a combined chemo-radiotherapy approach [32].

In patients with advanced-stage disease (III-IV), the therapeutic strategy depends on the tumor burden. In patients with a low tumor burden and asymptomatic disease, a watch-and-wait approach is often preferred, as several randomized studies have not demonstrated a survival benefit with early treatment [63,64].

This therapeutic strategy is grounded in the distinct biological and clinical characteristics of FL, which are typically associated with an indolent clinical course and relatively slow disease progression [63].

In asymptomatic patients with a low tumor burden, the immediate initiation of therapy does not confer a survival advantage compared to deferred treatment, and early therapy may expose patients to unnecessary toxicities, such as cytopenias, infections, and secondary malignancies, without improving long-term outcomes [63].

Therefore, observation is favored to preserve quality of life without compromising the prognosis. In recent years, the approach to managing low-tumor-burden FL has evolved to include functional imaging techniques, such as 18F-FDG PET/CT, and molecular profiling of MRD and key genetic mutations (e.g., TP53, KMT2D, and EZH2), which allow for a more individualized and refined risk stratification [64].

These advances assist in better tailoring the decision to defer or initiate therapy based on the biological and metabolic features predictive of disease aggressiveness. However, these patients require close monitoring [63,64].

In patients with advanced-stage disease and a high tumor burden, treatment initiation should follow the criteria established by GELF (Groupe d’Étude des Lymphomes Folliculaires) or BNLI (British National Lymphoma Investigation) [63,65]. The standard therapeutic options include immunochemotherapy regimens. Historically, the most commonly used treatment in fit patients has been R-bendamustine (RB), as demonstrated by the STiL trial, which showed superior PFS compared to R-CHOP, without impacting OS [66]. The BRIGHT trial also confirmed the non-inferiority of RB compared to R-CHOP and R-CVP [67]. However, R-CHOP remains a valid alternative, with the FOLL05 trial reporting 3-year PFS rates of 63% for R-CHOP, 52% for R-CVP, and 68% for R-FM (rituximab, fludarabine, and mitoxantrone). Despite its efficacy, grade 3–4 neutropenia was more frequent with R-FM, as well as a higher incidence of secondary malignancies [68].

Although RB has historically been one of the frontline regimens for FL, emerging data in the CAR-T era have raised important immunological concerns. Bendamustine induces a profound and prolonged depletion of CD4+ and CD8+ T-cells, with immune dysfunction persisting beyond one year. This immunosuppression compromises CAR T-cell expansion and persistence, increasing the risk of therapeutic failure. Furthermore, bendamustine upregulates inhibitory checkpoints such as PD-1 and LAG-3, promoting T-cell exhaustion. Thus, a careful selection of first-line therapy is essential, especially in patients potentially eligible for future CAR-T treatment [66].

In recent years, the use of obinutuzumab, an anti-CD20 type II monoclonal antibody, has increased. The GALLIUM trial demonstrated an improvement in the PFS of patients treated with obinutuzumab compared to rituximab (80% vs. 73% at 3 years), although no benefit in OS was observed. However, its toxicity profile is more severe, with a higher incidence of infectious complications and secondary malignancies, particularly in patients treated with bendamustine [69].

An alternative to chemotherapy is the R^2^ regimen (lenalidomide + rituximab), which represents a major advancement in the chemo-free management of FL, leveraging the immunomodulatory effects of lenalidomide to synergize with the anti-CD20 action of rituximab. Lenalidomide enhances ADCC by activating NK cells and augments T-cell function by promoting a Th1 cytokine response and reducing regulatory T-cell (Treg) populations [70]. This mechanism underpins its efficacy in FL, where immune evasion and microenvironmental support are critical for lymphoma survival.

The RELEVANCE trial, a landmark randomized Phase III study, evaluated R^2^ against standard chemoimmunotherapy (R-CHOP, R-CVP, or R-bendamustine) as the frontline treatment in patients with previously untreated advanced-stage FL. Although the primary endpoint of superiority was not met, R^2^ demonstrated non-inferior complete response (CR) rates (48% vs. 53%) and comparable 3-year PFS rates (77% vs. 78%), while offering a more favorable toxicity profile. Hematologic toxicities, particularly grade 3–4 neutropenia (31% vs. 50%) and febrile neutropenia (2% vs. 6%), were significantly lower in the R^2^ arm. Additionally, patients treated with R^2^ exhibited lower rates of secondary malignancies compared to those receiving chemotherapy, suggesting a safer long-term safety profile [70].

To evaluate the effectiveness of a response-adapted postinduction treatment in FL patients who responded to ICT, the FOLL12 prospective, randomized, open-label, multi-center Phase III trial was carried out by the Fondazione Italiana Linfomi. The study showed that, with a hazard ratio of 1.92 and 3-year PFS of 86% vs. 72%, the response-adapted postinduction therapy significantly underperformed standard maintenance therapy. The majority of subgroups, and especially patients with the best quality of response, as shown by both CMR and MRD negativity, showed inferiority of the response-adapted experimental arm. These findings lead to the conclusion that in order to ensure the lowest risk of lymphoma progression, patients with FL responding to induction ICT should be administered 2-year R maintenance therapy [32].

In the relapsed/refractory (R/R) setting, the AUGMENT trial firmly established the role of R^2^. In this double-blind Phase III study, R^2^ significantly improved the median PFS (39.4 months vs. 14.1 months) and overall response rate (78% vs. 53%) compared to rituximab monotherapy, demonstrating consistent benefits across multiple subgroups, including patients with early relapse (POD-24), bulky disease, and high FLIPI scores. The toxicity profile observed in AUGMENT was manageable, with neutropenia (32% grade ≥ 3), infections (9% grade ≥ 3), and rash (7% grade ≥ 3) as the most common adverse events [71]. Importantly, R^2^ therapy was associated with the preservation of immune effector cells (T cells and NK cells), an important consideration in the era of immunotherapeutic strategies such as CAR-T [72].

Collectively, these data support the integration of R^2^ as an effective, chemo-free option in both frontline and relapsed settings, particularly for patients who seek to minimize the long-term immunosuppressive effects of conventional chemotherapy and preserve eligibility for future immune-based interventions [70,72].

In frail patients or those with significant comorbidities, the use of rituximab monotherapy represents a valid therapeutic strategy [64]. The RESORT study randomly assigned patients with a low tumor burden who had achieved a partial response (PR) or CR after four weekly doses of rituximab to two treatment strategies: rituximab maintenance or observation, with the option of retreatment upon disease progression. The primary endpoint, time to treatment failure, showed no significant differences between the two groups [73].

After induction therapy, FDG-PET can serve as a valuable prognostic tool to identify complete metabolic responses and guide potential maintenance strategies [71,74,75].

The PRIMA trial demonstrated that rituximab maintenance therapy for 2 years prolongs PFS (75% vs. 58%) compared to observation alone, although it is associated with a higher risk of infections [76].

Although first-line therapies for FL achieve high response rates, most patients eventually experience disease relapse. A crucial prognostic factor is the time to disease progression: approximately 20% of patients treated with chemoimmunotherapy develop POD-24, a condition associated with a significantly reduced OS [57]. Histological confirmation of transformation into a more aggressive lymphoma, such as DLBCL, is essential in patients with early progression, as they appear to have a higher risk of transformation [77].

In patients with R/R FL, therapeutic options vary depending on the clinical presentation. Asymptomatic patients can be managed with observation, while in cases of localized relapse, radiotherapy remains a valid option. When systemic treatment is required, rituximab monotherapy or chemoimmunotherapy are well-established strategies, with rituximab retreatment being particularly effective in patients who achieved a prolonged remission with first-line therapy [73].

For rituximab-refractory patients, the use of obinutuzumab, as demonstrated in the GADOLIN study, has shown an improvement in OS when combined with bendamustine [78]. Rituximab maintenance therapy can be considered for patients who achieve a partial or complete response after second-line therapy, as it has been associated with prolonged PFS compared to observation alone. However, the benefit for OS remains uncertain [79].

Autologous stem cell transplantation (ASCT) remains an option for patients with chemosensitive disease, particularly those with POD-24, in whom ASCT consolidation may improve disease control. Retrospective studies have suggested an OS benefit, but the role of transplantation has declined with the introduction of novel targeted therapies and immunotherapies. Substantial differences exist between Europe and the United States regarding ASCT utilization. In Europe, ASCT is routinely considered for high-risk FL and transformed cases, mainly when CAR-T therapies are not accessible. Conversely, in the United States, the broader availability and earlier integration of CAR-T therapy into practice have significantly reduced ASCT use, reserving it for patients who relapse after CAR-T or those ineligible for cellular therapies [80]. These variations reflect differences in healthcare system organization, regulatory approvals, and treatment philosophy.

In patients who relapse after CAR-T therapy or BsAbs, allogeneic stem cell transplantation (allo-SCT) remains a potential option, especially in settings where these therapies are not available [80,81,82]. The treatment landscape for R/R FL is continuously evolving with the introduction of novel targeted agents. Among these, lenalidomide, an immunomodulatory agent [70,72], and epigenetic inhibitors, such as tazemetostat, have demonstrated promising efficacy, particularly in patients with EZH2 mutations, achieving an overall response rate (ORR) of 69% and a median PFS of over 13 months [83]. Additionally, PI3K inhibitors (idelalisib, duvelisib, and copanlisib) have shown efficacy in patients with R/R FL; however, their use is limited due to high toxicity [84,85,86].

Among the new immunotherapies, BsAbs and CAR-T therapy are revolutionizing the treatment of FL. BsAbs, such as mosunetuzumab, glofitamab, and epcoritamab, activate T cells against malignant B cells, demonstrating high response rates even in high-risk patients, including those with POD-24. Clinical trials have reported ORRs exceeding 78% and a median PFS of over 18 months, with a favorable safety profile compared to CAR-T therapy, due to significantly lower rates of cytokine release syndrome (CRS) and immune effector cell-associated neurotoxicity syndrome (ICANS) [87,88,89,90].

CAR-T cell therapy has revolutionized the management of R/R FL, providing durable disease control even in heavily pretreated populations. Two pivotal Phase II studies, ZUMA-5 and ELARA, have defined the efficacy and safety profile of CAR-T therapy in this setting [91,92,93].

In ZUMA-5, axicabtagene ciloleucel (Axi-cel) achieved an ORR of 94% and a CR rate of 79%, with a 3-year PFS of over 50% and a 3-year OS of 84%, demonstrating sustained long-term benefits. These outcomes were consistent across high-risk subgroups, such as those with POD-24 or bulky disease. CRS ≥ grade 3 occurred in 6% and neurotoxicity in 15%, which were manageable with current supportive measures [89,90,91].

Similarly, ELARA demonstrated that tisagenlecleucel (Tisa-cel) achieved an ORR of 86% and CR of 69%, with 2-year PFS rates exceeding 57%. Tisa-cel’s safety profile was remarkably favorable, with no grade ≥ 3 CRS events, making it attractive for older or comorbid patients [88,89,92,93].

Importantly, CAR-T therapy has also shown significant efficacy in tFL. In ZUMA-5, tFL patients achieved ORRs and CR rates comparable to de novo FL, suggesting that CAR-T therapy can overcome the adverse molecular features associated with transformation [91,92].

The overall favorable safety profile of CAR-T in FL compared to aggressive lymphomas supports its broader application. Ongoing clinical trials aim to evaluate CAR-T therapy earlier in the treatment algorithm, potentially after the first relapse, and in combination with novel immunotherapies to further enhance the efficacy and durability of responses [87,88,89,90].

Phase II studies, including ZUMA-5 and ELARA, have reported a median PFS of over 40 months with Axi-cel and a 2-year PFS rate of 57% with Tisa-cel, making this strategy particularly promising for patients with early relapse or chemorefractory disease [91,92,93].

With the advancement of targeted and immunotherapies, the treatment of R/R FL is increasingly shifting toward personalized approaches, aiming to prolong the response duration and improve quality of life, while minimizing the toxicity associated with traditional chemotherapy.

In Table 2, we summarize the mechanisms, advantages, and toxicities of mAbs, BsAbs, and ADCs usable for FL.

### 2.3. Focus on Approved mAbs

mAbs selectively target antigens expressed on malignant B cells, thereby enhancing immune-mediated cytotoxicity and reducing the tumor burden. Among these, anti-CD20 antibodies have significantly improved patient outcomes, both as monotherapy and in combination with chemotherapy [69,94]. This section explores the three currently approved monoclonal antibodies for FL treatment, rituximab, obinutuzumab, and mosunetuzumab, highlighting their mechanisms of action, clinical efficacy, and therapeutic implications.

#### 2.3.1. Rituximab

Rituximab (MabThera^®^/Rituxan^®^/Rixathon^®^/Truxima^®^/Ruxience^®^/Rituxan HYCELA™) is the first anti-CD20 mAb that has revolutionized the treatment of FL. It is a chimeric IgG1-κ antibody, consisting of a murine variable region and a human constant region, which binds with high affinity to CD20, a transmembrane antigen expressed on the surface of normal and malignant mature B cells. CD20 binding triggers a series of B-cell elimination mechanisms, including ADCC, phagocytosis, CDC, and direct apoptosis [95,96,97]. Complement activation is a hallmark of type I anti-CD20 antibodies, to which rituximab belongs, distinguishing them from type II antibodies such as obinutuzumab, which exhibit a different mechanism of action [98].

Its pharmacokinetic (PK) profile has been investigated in numerous clinical trials, showing detectable plasma concentrations after the first infusion, with its levels progressively increasing with subsequent administrations [99,100,101]. In patients with low-grade lymphoma treated with four weekly infusions (375 mg/m^2^), peak serum concentrations (Cmax) ranged from 77.5 to 996.6 µg/mL after the fourth dose, with the drug remaining detectable in serum for up to six months after the last administration [94,102]. Rituximab elimination follows a target-mediated mechanism dependent on CD20 binding, leading to an initially rapid clearance, followed by a progressive reduction as circulating B cells are depleted [101]. Furthermore, population pharmacokinetic studies have demonstrated a correlation between serum rituximab concentrations and the clinical response, with higher levels observed in responders compared to non-responders [101,103]. The approval of rituximab in 1997 for the treatment of FL was based on data from a phase II study demonstrating an ORR of 48% in patients with R/R indolent NHL [104]. The combination of rituximab with standard chemotherapy regimens (CHOP, CVP, and FCM) significantly improved clinical outcomes compared to chemotherapy alone, leading to its adoption as the gold standard for the treatment of advanced FL [105,106,107,108,109]. The addition of chemotherapy resulted in higher response rates and a significant prolongation of PFS compared to monotherapy. The PRIMA trial later demonstrated that two years of maintenance rituximab therapy in responder patients after induction therapy with R-CHOP, R-CVP, or R-FCM improved PFS compared to observation [76].

In R/R FL, the efficacy of rituximab has been evaluated in several clinical trials, demonstrating that its addition to chemotherapy significantly improves clinical outcomes. For instance, the EORTC 20981 study reported an improvement in the median PFS (51.5 vs. 14.9 months; HR 0.40; *p* < 0.001) of patients receiving rituximab maintenance therapy compared to observation alone [79]. Additionally, a meta-analysis of seven randomized studies confirmed an improvement in OS with rituximab maintenance, except in patients who had already received rituximab during induction therapy [110].

Although generally well tolerated, rituximab is associated with infusion-related reactions, immunosuppression, and an increased risk of infections. Infusion-related reactions, such as fever, chills, and hypotension, are among the most common adverse events, while prolonged immunosuppression increases the risk of opportunistic infections, including hepatitis B virus reactivation, necessitating serological screening before initiating treatment [111,112,113]. Rare but serious adverse effects include progressive multifocal leukoencephalopathy (PML) caused by JC virus reactivation, which is associated with a poor prognosis [114]. Certain patient subpopulations, such as elderly individuals and those with bulky disease, may experience a higher incidence of grade 3–4 adverse events. In patients with chronic lymphocytic leukemia (CLL), pre-infusion prednisone is recommended to reduce the risk of cytokine release syndrome, while those with a high tumor burden require adequate hydration and prophylaxis for tumor lysis syndrome [111,115].

Despite the emergence of new immunotherapies, rituximab remains a cornerstone in the treatment of FL. Its efficacy has been demonstrated in numerous clinical trials, both as monotherapy and in combination with chemotherapy, solidifying its role in clinical practice. The evolution of therapeutic strategies is leading to an increasingly targeted use of the drug, aiming to maximize the clinical benefit while minimizing toxicity, particularly in more vulnerable patient populations.

#### 2.3.2. Obinutuzumab

Obinutuzumab (Gazyva^®^/Gazyvaro^®^) is a third-generation humanized anti-CD20 monoclonal antibody developed using glycoengineering techniques to enhance its therapeutic activity [116]. Unlike rituximab, which belongs to the type I antibody category, obinutuzumab is a type II antibody, characterized by a different binding orientation to CD20 and a greater ability to induce ADCC and antibody-dependent cellular phagocytosis (ADCP) while exhibiting reduced complement activation [117,118]. Due to these features, obinutuzumab promotes an increased level of direct non-apoptotic cell death, distinguishing it from type I mAbs such as rituximab and ofatumumab [119].

Phase I and II studies, such as GAUGUIN, have helped establish the optimal administration regimen, demonstrating that a higher loading dose during the initial administrations allows for a more rapid achievement of effective plasma concentrations, enhancing B-cell depletion and disease control [120,121]. The clinical efficacy of obinutuzumab has been demonstrated in several studies evaluating its use both as a monotherapy and in combination with chemotherapy. The Phase III GALLIUM study compared induction therapy with obinutuzumab versus rituximab, both administered with chemotherapy and followed by maintenance therapy, in patients with previously untreated FL. The results showed a significant improvement in the PFS of patients treated with obinutuzumab, with a 34% lower risk of progression, relapse, or death compared to the rituximab-treated group. However, no significant difference in OS was observed between the two groups, suggesting that the benefit of obinutuzumab lies primarily in long-term disease control rather than an increase in overall survival [69,122]. From a pharmacokinetic perspective, obinutuzumab differs from rituximab in its dosing regimen. While rituximab is administered based on the body surface area, obinutuzumab utilizes a fixed dosing approach to minimize interindividual variability [123,124].

In the treatment of R/R FL, the GADOLIN study demonstrated a significant improvement in the PFS of patients treated with obinutuzumab in combination with bendamustine compared to bendamustine monotherapy (25 vs. 14 months, *p* < 0.0001), along with a survival benefit (HR 0.58, *p* = 0.0061). Additionally, MRD negativity was significantly higher in patients treated with obinutuzumab, suggesting a deeper response [125].

Despite the improvement in clinical outcomes, the safety profile of obinutuzumab presents some concerns compared to rituximab. Grade 3–5 adverse events were more frequent in patients treated with obinutuzumab, particularly infusion-related reactions (IRRs) and neutropenia [121,123,126,127,128,129]. The increased incidence of IRRs has been attributed to higher cytokine release during the first infusion, with elevated levels of IL-6, IL-8, IL-10, TNF-α, and interferon-gamma compared to rituximab. However, these reactions can be mitigated with appropriate premedication and careful management of the infusion rate [69].

Another important consideration is the increased incidence of infections and secondary malignancies in patients treated with obinutuzumab, particularly when combined with bendamustine [130]. Concerning safety signals, besides infusion-related reactions and frequent infections, this medication is related to a risk of tumor lysis syndrome that can occur in patients with a high tumor burden. Additionally, like other anti-CD20 monoclonal antibodies, obinutuzumab can cause hepatitis B reactivation and, in rare cases, PML, necessitating pretreatment screening [131,132].

The future of obinutuzumab therapy may lie in its combination with novel targeted agents, such as lenalidomide, to optimize efficacy while reducing toxicity. The rationale for combining obinutuzumab with lenalidomide for follicular lymphoma lies in their synergistic immunomodulatory effects: since obinutuzumab targets CD20 on B cells, enhancing antibody-dependent cellular cytotoxicity, and lenalidomide boosts the immune response by activating T cells and NK cells, the result is an improvement in anti-tumor activity without relying on conventional chemotherapy. The chemo-free nature of this combination makes it particularly appealing for patients unsuitable for traditional chemotherapy. Overall, it represents a promising strategy in the evolving treatment landscape of follicular lymphoma.

#### 2.3.3. Mosunetuzumab

**Figure 1:**
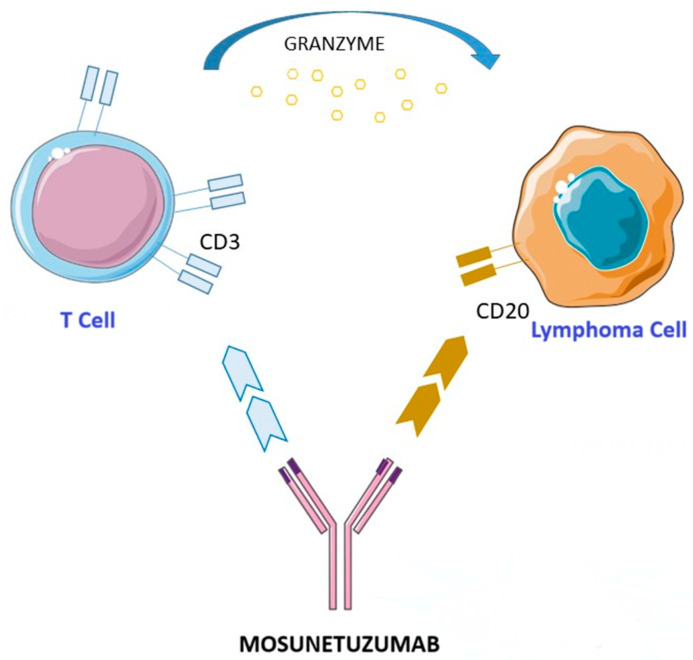
Mechanism of action of mosunetuzumab. This figure was partly generated using Servier Medical Art, provided by Servier, licensed under a Creative Commons Attribution 3.0 Unported License.

Mosunetuzumab (Lunsumio™) (Figure 1) is a bispecific anti-CD20/CD3 antibody belonging to the class of T-cell engagers developed to redirect cytotoxic T cells toward malignant B cells, thereby promoting a targeted immune system activation. Unlike traditional monoclonal antibodies, mosunetuzumab is designed to simultaneously bind CD20, which is expressed on malignant B cells, and CD3, which is present on T cells, inducing specific activation and a direct cytotoxic action against tumor cells. This therapeutic strategy enables a complement-independent mechanism of action, reducing the risk of resistance observed with conventional anti-CD20 therapies. Moreover, its full-length humanized IgG1 structure, which was developed using knobs-into-holes technology, provides an extended half-life compared to other BsAbs, eliminating the need for continuous infusion [133,134,135].

The efficacy of mosunetuzumab was evaluated in the Phase I/II GO29781 study, which included patients with R/R FL and DLBCL who had failed at least two prior lines of therapy. Patients were treated with a step-up dosing regimen to mitigate the risk of CRS: 1 mg on day 1, 2 mg on day 8, and 60 mg on day 15 of the first cycle, followed by 60 mg in cycle 2 and 30 mg from cycle 3 onward. The ORR was 80%, with a CR in 60% of patients, and the median PFS was not reached. Positive outcomes were also observed in heavily pretreated patients, including those refractory to both an anti-CD20 agent and an alkylating agent (double-refractory), as well as those with POD-24 [88,136,137].

The PK profile of mosunetuzumab was studied in the Phase II cohort of the GO29781 trial, demonstrating prolonged drug availability, with detectable serum levels after the first administration. The sequential step-up dosing regimen optimized drug exposure while minimizing the risk of CRS, which occurred in 27% of patients (primarily grade 1–2), with only 2% experiencing grade ≥3 CRS. The most common adverse events included neutropenia (28.4%), anemia (18.8%), and hypophosphatemia (15.2%), while the incidence of severe infections (grade ≥ 3) was 14%, which was comparable to other anti-CD20 therapies. Only 4% of patients discontinued treatment due to adverse effects, highlighting an overall favorable tolerability profile [88].

Mosunetuzumab has received FDA and EMA approval for the treatment of R/R FL after at least two prior lines of therapy, based on the efficacy and safety data from the GO29781 trial. A comparison with other available therapeutic options, R2 and tazemetostat (EZH2i), has shown that mosunetuzumab offers a higher CR rate and sustained efficacy even in patients with refractory disease [72,83,85,86,138,139]. A comparison with other available therapeutic options, such as PI3K inhibitors (duvelisib, umbralisib, and copanlisib), R^2^, and tazemetostat, has shown that mosunetuzumab offers a higher CR rate and sustained efficacy even in patients with refractory disease [72,83,85,86,138,139].

Compared to CAR-T therapies (Axi-cel and Tisa-cel), mosunetuzumab stands out for its simpler administration and a more favorable toxicity profile. In the ZUMA-5 and ELARA trials, the incidence of severe CRS (≥6%) and ICANS (≥3%) was higher with CAR-T therapy than with mosunetuzumab. Additionally, the need to wait weeks for CAR-T cell manufacturing and its administration being restricted to specialized centers present logistical challenges that mosunetuzumab can overcome, offering an immediately available treatment that may be administered in an outpatient setting [88,92,93].

The evolving therapeutic resources have led to the initiation of multiple combination clinical trials aimed at optimizing the use of mosunetuzumab. Ongoing Phase II and III studies are currently evaluating its combination with lenalidomide (NCT04792502, NCT04246086, and NCT06284122), tazemetostat (NCT05994235), zanubrutinib (NCT06492837), and polatuzumab vedotin (NCT05410418 and NCT06453044). Mosunetuzumab represents a valuable therapeutic innovation in the treatment of FL, offering high efficacy and better tolerability compared to CAR-T therapies. If ongoing studies confirm the preliminary results, its use could be extended to patients with earlier-stage disease, establishing itself as an innovative and promising strategy to improve FL management.

A comparative table summarizing the main mechanistic and clinical features of the mAbs discussed is reported below (Table 3).

### 2.4. Ongoing Clinical Trials

In the past two years, multiple Phase I–III clinical trials have significantly reshaped the treatment paradigm of FL through the development of novel monoclonal antibodies, particularly CD20/CD3 BsAbs and ADCs, offering highly effective, chemotherapy-free alternatives [140] (Table 4).

Among these, the Phase I/II NP30179 trial evaluating glofitamab, a “2:1” BsAbs that engages two CD20 epitopes and one CD3 domain, has demonstrated a remarkable ORR of 81% and a CR rate of 70%specifically in heavily pretreated R/R FL patients, many of whom had received multiple prior therapies [141]. The median duration of response (DOR) exceeded 24 months, with long-term remission observed in a substantial proportion of patients, suggesting the potential for prolonged disease control [147]. CRS was the predominant adverse event, occurring in 66% of patients, but primarily as low-grade (grade 1–2) events; only 3% experienced grade ≥ 3 CRS, mitigated by an optimized step-up dosing regimen that included the pre-administration of obinutuzumab to reduce excessive T-cell activation and immune-mediated toxicity [148].

Similarly, epcoritamab, a subcutaneously administered CD20 × CD3 BsAbs, has shown promising activity in the Phase II EPCORE NHL-1 trial, enrolling patients with advanced-stage and heavily pretreated R/R FL. The study achieved an ORR of 82.0% and a CR rate of 62.5% in patients with advanced FL [142]. Importantly, epcoritamab demonstrated an improved safety profile compared to intravenous BsAbs, with grade ≥ 3 CRS occurring in only 2% of patients [90]. The subcutaneous delivery method may contribute to its lower CRS incidence and improved tolerability, positioning it as a strong candidate for broader clinical use [149].

The Phase II ELM-2 trial of odronextamab, another CD20 × CD3 BsAbs, included R/R FL patients with a history of at least two prior systemic therapies, reflecting a population with high unmet clinical needs. This trial further supports the transformative potential of this class, with an ORR of 82% and a CR of 75% in heavily pretreated FL patients [143,150]. Notably, the median PFS was approximately 20 months, reinforcing the durability of responses with T-cell-engaging therapies [151]. CRS was observed in 56% of patients, yet severe events were rare (~2% grade ≥ 3 CRS) due to a step-up dosing protocol designed to prevent severe ICANS, which was notably absent in the study cohort [152,153]. In parallel with BsAbs, anti-CD19-directed therapies have emerged as a key area of interest, aiming to target residual malignant B cells in R/RFL [154]. The Phase III inMIND trial evaluated tafasitamab-cxix, an Fc-enhanced anti-CD19 monoclonal antibody, in combination with R^2^ in patients with relapsed or refractory FL who had received between one and three prior lines of therapy. The study demonstrated a significant improvement in PFS compared to R^2^ alone, successfully meeting its primary endpoint while maintaining a favorable safety profile with no novel toxicity signs [144].

Tafasitamab’s role in FL appears particularly promising, as its mechanism of action—enhancing ADCC and macrophage-mediated phagocytosis—complements existing immunomodulatory regimens, potentially augmenting the response depth and durability beyond standard anti-CD20 therapies [145]. Another emerging anti-CD19 therapy, loncastuximab tesirine, an ADC delivering a cytotoxic pyrrolobenzodiazepine payload, was evaluated in the Phase II LOTIS-5 trial [146]. This study targeted a high-risk R/R FL population, including a significant proportion of patients with POD-24, a group with historically poor outcomes. The combination of loncastuximab tesirine and rituximab achieved a striking ORR of 97% and a CR rate of 77% in high-risk R/R FL patients, with a 12-month PFS rate of 95%, demonstrating a highly durable response profile in a heavily pretreated population [155,156]. While the treatment was generally well tolerated, hematologic toxicity and hepatotoxicity required close monitoring, particularly in frail patients, reflecting the necessity of patient selection for ADC-based regimens [157].

The integration of these novel monoclonal antibody-based therapies into the FL treatment is poised to significantly improve patient outcomes [158]. BsAbs are increasingly being positioned as alternatives to conventional chemotherapy, with mounting evidence supporting their use as both monotherapy and in combination regimens, particularly for patients with high-risk disease features [159]. The success of anti-CD19 ADCs suggests that combining direct B-cell cytotoxicity with immune-based approaches may further enhance treatment efficacy, particularly in patients with POD-24 disease who have a poor prognosis under conventional treatment paradigms [160].

As ongoing Phase III trials such as EPCORE FL-1, ELM-4, and inMIND evaluate these agents in earlier treatment lines, the positioning of BsAbs and ADCs is expected to shift toward frontline and second-line settings, potentially redefining the standard of care in FL by offering deep, durable responses with a reduced reliance on cytotoxic chemotherapy [161]. These findings collectively indicate that monoclonal antibody therapies are ushering in a new era in FL treatment, shifting toward highly targeted, immune-mediated, and potentially curative strategies.

In the next table, we summarize key clinical trials with the patient populations, designs, outcomes, and limitations in treating FL (Table 5).

## 3. Challenges and Future Directions

Despite the significant advances in monoclonal antibody therapies for FL, several challenges remain that must be addressed to optimize long-term patient outcomes. One critical area involves the personalization of treatment through biomarker-driven selection. Currently, therapeutic decisions are rarely based on molecular or immune profiling, yet emerging evidence suggests that factors such as EZH2 mutations, TP53 alterations, CD20 expression levels, and the immune composition of the tumor microenvironment could inform the optimal use of monoclonal antibodies. Incorporating these biomarkers, alongside the MRD status, into prospective clinical trials could significantly enhance treatment precision [26,35].

Another pressing issue concerns the cost-effectiveness and accessibility of these agents. While rituximab is now widely available as a biosimilar, next-generation mAbs, BsAbs, and CAR-T therapies remain costly and often inaccessible in low- and middle-income settings. Addressing these disparities will require not only pharmacoeconomic analyses but also policy-level efforts to facilitate equitable access to novel immunotherapies [2,3,4].

In parallel, the optimal sequence of antibody-based therapies remains unresolved. The growing number of available options—including conventional mAbs, BsAbs, and CAR-T cells—raises important questions about how to integrate and prioritize these treatments across different disease stages and patient profiles. BsAbs offer advantages such as lower toxicity and outpatient administration, whereas CAR-T therapy provides long-lasting remission in high-risk populations. Evidence-based frameworks to guide sequencing strategies will be essential in clinical practice.

Finally, therapeutic resistance remains a major obstacle. CD20 antigen loss, immune evasion, and T-cell exhaustion contribute to both primary and acquired resistance to antibody-based therapies. Although BsAbs partly overcome these limitations by directly engaging T cells, resistance mechanisms may still arise, particularly in immunosuppressive microenvironments [88,140,159]. Ongoing trials investigating combination strategies with immune checkpoint inhibitors, epigenetic modulators, and cytokine agonists represent promising avenues to restore antitumor immunity and improve therapeutic durability.

## 4. Conclusions

In conclusion, the development and application of monoclonal antibodies, particularly those targeting CD20, have substantially transformed the treatment of FL, offering new hope for patients through targeted therapies that improve clinical outcomes and reduce systemic toxicity [10,51,63].

The continued exploration of combination therapies and optimization of treatment regimens will play a crucial role in enhancing the effectiveness of monoclonal antibodies [7,61].

Future prospects in the treatment of FL are increasingly promising due to advancements in personalized medicine and targeted therapies. The use of genomic profiling enables clinicians to customize treatment plans based on individual tumor characteristics. Novel agents like CAR-T cell therapy, BsAbs, and small-molecule inhibitors are showing effectiveness, especially in relapsed or refractory cases. Immunotherapy continues to evolve, aiming to enhance the immune response while reducing toxicity. Ongoing clinical trials are crucial to developing more durable and potentially curative treatments.

## Figures and Tables

**Table 1:** Prognostic systems and corresponding outcomes of FL.

Index	Year	Parameters	Risk Groups	Outcomes	References
**FLIPI**	2004	Age > 60 years Stage III/IV Hemoglobin < 12 g/dL >4 nodal areas Elevated LDH levels	Low (0–1), Intermediate (2), High (3–5)	10-year Overall Survival (OS): Low 70%, Intermediate 50%, High 35%	[50,51,52]
**FLIPI-2**	2009	Age > 60 β2-microglobulin > normal Longest node > 6 cm Bone marrow involvement Hemoglobin < 12 g/dL	Low (0–1), Intermediate (2), High (3–5)	5-year Progression-Free Survival (PFS): Low 79%, Intermediate 51%, High 19%	[50,51,52]
**m7-FLIPI**	2015	FLIPI ECOG Performance Status Mutation status of 7 genes: EZH2, ARID1A, MEF2B, EP300, FOXO1, CREBBP, and CARD11	Low vs. high Risk	5-year PFS: Low Risk ~77%, High Risk ~30%	[50,51,52]

**Table 2:** Mechanisms, advantages, and toxicities of mAbs, BsAbs, and ADCs in FL.

Therapy Class	Mechanism of Action	Advantages	Common Toxicities	References
**Monoclonal Antibodies**	Bind to CD20 on B cells and induce immune-mediated cytotoxicity	-Established efficacy -Well-tolerated -Synergistic with chemotherapy	-Infusion-related reactions -Risk of infections -Neutropenia	[2]
**Bispecific Antibodies**	Bind both CD20 on B cells and CD3 on T cells and redirect T-cell cytotoxicity against lymphoma cells	-Off-the-shelf -Effective against R/R FL -No need for patient-specific manufacturing	-CRS -ICANS -Infections	[3,4]
**Antibody–Drug Conjugates**	The antibody binds the target and, after internalization, there is the release of the cytotoxic payload and cell death	-Targeted delivery of chemotherapy -Reduced systemic toxicity -Activity in resistant cases	-Peripheral neuropathy -Myelosuppression -Diarrhea	[11]

**Table 3:** Comparison of the main mechanistic and clinical features of rituximab, obinutuzumab, and mosunetuzumab in treating follicular lymphoma.

Feature	Rituximab	Obinutuzumab	Mosunetuzumab
**Type**	Chimeric anti-CD20 IgG1 (Type I)	Humanized anti-CD20 IgG1 (Type II)	Bispecific anti-CD20/CD3 IgG1
**Mechanism of Action**	ADCC, CDC, direct apoptosis	Enhanced ADCC, direct cell death, reduced CDC	T-cell redirection, independent of CDC/MHC
**Structure**	Chimeric (murine/human)	Glycoengineered humanized antibody	Full-length humanized bispecific antibody
**Clinical Indication**	First-line and relapsed/refractory FL	First-line and relapsed/refractory FL	Relapsed/refractory FL after ≥2 prior therapies
**Efficacy**	Improved PFS and OS with chemoimmunotherapy	Superior PFS vs. rituximab (GALLIUM trial)	High ORR and CR in heavily pretreated patients (GO29781 study)
**Main Toxicities**	Infusion reactions, infections, rare PML	Higher infusion reactions, neutropenia, infections	Cytokine release syndrome (mild/moderate), neutropenia
**Administration**	Intravenous or subcutaneous	Intravenous	Step-up intravenous dosing (outpatient feasible)
**References**	[95,96,97,98,99,100,101,102,103,104,105,106,107,108,109,110,111,112,113,114,115]	[116,117,118,119,120,121,122,123,124,125,126,127,128,129,130,131,132]	[88,133,134,135,136,137,138,139]

**Table 4:** Summary of studies on CD20/CD3 BsAbs and ADC in follicular lymphoma.

**Drug**	**Study Title**	**Object of the Study**	**Main Outcomes**	**Reference**
**Glofitamab**	**Phase I/II NP30179 trial**	heavily pretreated R/R FL patients	ORR of 81% and CR rate of 70%	[141]
**Epcoritamab**	**Phase II EPCORE NHL-1**	patients with advanced FL	ORR of 82.0% and CR rate of 62.5%	[142]
**Odronextamab**	**Phase II ELM-2 trial**	heavily pretreated FL patients	ORR of 82% and a CR of 75%	[143]
**Tafasitamab**	**Phase III in MIND trial**	residual malignant B cells in FL	A significant improvement in PFS compared to R^2^ alone, successfully meeting its primary endpoint while maintaining a favorable safety profile with no novel toxicity signals	[144,145]
**Loncastuximab and rituximab**	**Phase II LOTIS-5 trial**	heavily pretreated FL patients	ORR of 97% and a CR rate of 77% in high-risk R/R FL patients, with a 12-month PFS rate of 95%	[146]

**Table 5:** Key clinical trials with patient populations, design, outcomes, and limitations in treating follicular lymphoma.

Trial	Patient Population	Design	Key Outcomes	Limitations	References
**GALLIUM**	Previously untreated FL (Grade 1–3a)	Phase III, randomized obinutuzumab + chemotherapy vs. rituximab + chemotherapy → anti-CD20 maintenance therapy	The obinutuzumab arm exhibited an improved PFS at 7 years (63.4% vs. 55.7%). No OS difference.	Higher toxicity (e.g., neutropenia and infections) No OS benefit	[122]
**GADOLIN**	Rituximab-refractory indolent NHL (incl. FL)	Phase III, randomized obinutuzumab + bendamustine vs. bendamustine alone	Median PFS: 29.2 vs. 13.7 months. OS benefit observed.	Rituximab-refractory population only Potential selection bias	[125]
**ZUMA-5**	R/R FL and MZL after ≥ 2 prior lines	Phase II, single-arm Axi-cel CAR-T cell therapy	ORR of 92% and CR of 76% in patients with FL. Durable remissions with 2-year PFS of ~60%.	Single-arm, no comparator toxicity: CRS and ICANS	[92]
**RELEVANCE**	Treatment-naïve FL	Phase III, randomized R^2^ (rituximab + lenalidomide) vs. chemoimmunotherapy	Similar PFS and OS. Non-inferior but not superior.	Did not meet superiority The long-term safety of R^2^ is still under study	[4]
**ELARA**	R/R FL after ≥ 2 prior therapies	Phase II, single-arm tisagenlecleucel (CAR T)	ORR of 91%, CR of 75%, 1-year PFS ~70%	No control arm CAR T toxicities require monitoring	[4]
